# An online home-based exercise program improves autonomic dysfunction in breast cancer survivors

**DOI:** 10.3389/fphys.2023.1256644

**Published:** 2023-09-29

**Authors:** Ana Myriam Lavín-Pérez, Daniel Collado-Mateo, Carmen Hinojo González, Marco Batista, Xián Mayo, Cristina Ruisánchez Villar, Alfonso Jiménez

**Affiliations:** ^1^ Sport Sciences Research Centre, Rey Juan Carlos University, Madrid, Spain; ^2^ GO Fit LAB, GO Fit Life, Science and Technology, S.A., Madrid, Spain; ^3^ Program of Epidemiology and Public Health (Interuniversity), Ph.D. International School of Rey Juan Carlos University, Madrid, Spain; ^4^ Oncology Department, Hospital Universitario Marques de Valdecilla, Santander, Spain; ^5^ Sport, Health, and Exercise Research Unit (SHERU), Polytechnic Institute of Castelo Branco, Castelo Branco, Portugal; ^6^ Cardiology Department, Hospital Universitario Marqués de Valdecilla, Santander, Spain; ^7^ Advanced Wellbeing Research Centre, College of Health, Wellbeing and Life Sciences, Sheffield Hallam University, Sheffield, United Kingdom

**Keywords:** training program, autonomic dysfunction, breast cancer, heart rate variability, treatments cardiotoxicity

## Abstract

**Introduction:** Exercise interventions for breast cancer survivors have proved their potential to improve clinical, physical, and psychosocial outcomes. However, limited studies have explored exercise effects on autonomic dysfunction and the measurement of exercise tolerance and progression through daily heart rate variability (HRV).

**Purpose:** To analyze the effects of a 16-wk exercise intervention on the autonomic modulation of breast cancer survivors, as well as to examine the evolution of daily measured HRV and its interaction with exercise sessions in this population.

**Methods:** A total of 29 patients who had undergone chemotherapy and radiotherapy were randomly assigned to the exercise group or to the control group. The exercise intervention was delivered remotely through online meetings and consisted of supervised training resistance and cardiovascular exercise 3 times per week. During the intervention all patients measured their HRV daily obtaining the napierian logarithm of the root mean square of successive differences between normal heartbeats (lnrMSSD) and the napierian logarithm of the standard deviation of the interbeat interval of normal sinus beats (lnSDNN) values at four moments: day 0 (the morning of the training sessions), 24, 48, and 72 h after exercise.

**Results:** The results revealed a significant interaction between group and months during the intervention period for lnrMSSD and lnSDNN (*p* < 0.001). Additionally, there were significant differences in lnSDNN recovery time between months (*p* < 0.05), while differences in lnrMSSD become apparent only 24 h after exercise (*p* = 0.019). The control group experienced a significant decrease in both variables monthly (*p* < 0.05) while exercise group experienced a significant increment (*p* < 0.05).

**Conclusion:** HRV is daily affected by exercise training sessions in cancer patients. Although results strongly support the role of exercise as a post-chemotherapy and radiotherapy rehabilitation strategy for breast cancer survivors to improve autonomic imbalance, further research is necessary to validate these initial findings.

## 1 Introduction

The prevalence of breast cancer is constantly growing, and advances in research have led to increasing survival rates (Giaquinto et al., 2022). It is expected that by 2040, the number of new diagnosed cases of breast cancer will increase by more than 40%, reaching about 3 million cases annually (Arnold et al., 2022). Similarly, deaths from breast cancer are expected to increase by more than 50%, from 685,000 in 2020 to 1 million in 2040 (Arnold et al., 2022). Consequently, breast cancer survivors have a higher likelihood of experiencing the long-term effects of cancer and its treatments (Di Nardo et al., 2022). In this regard, research on breast cancer and effective therapies to mitigate treatment side effects has increased exponentially in recent decades (del-Rosal-Jurado et al., 2020).

Resistance and aerobic training during the survivorship phase can enhance clinical, physical, and psychosocial parameters (Ficarra et al., 2022) by improving physical fitness, muscle mass, and cardiopulmonary capacity (Lavín-Pérez A. M. et al., 2021), quality of life (Lavín-Pérez Ana et al., 2021), fatigue (Arnold et al., 2022), visceral adiposity (Garcia-Unciti et al., 2023), or joint pain (Lu, Zheng, and Zhang, 2020). However, one of the late side effects of treatments, on which exercise therapy is promising but scarce (Lee et al., 2019), is cardiac toxicity and autonomic dysfunction.

The cardiac toxicity resulting from medical therapies used to treat cancer can lead to various cardiovascular abnormalities such as myocarditis, heart failure, hypertension, arrhythmias, among others (Chang et al., 2017). Manifestations of cardiac toxicity may begin at the end of chemotherapy and radiotherapy treatments while its consequences may appear years after these treatments have ceased (Kirkham et al., 2019). In this regard, it can directly affect the development of comorbidities, survival rates, and quality of life of patients (Kirkham et al., 2019).Heart Rate Variability (HRV) assessment is a prognostic factor for cancer survival and has emerged as a crucial variable for detecting these adverse cardiovascular events even when the left ventricular function is within the reference range (Tjeerdsma et al., 1999). In this regard, breast cancer patients often exhibit an autonomic system imbalance due to this toxicity, resulting in autonomic dysfunction (Lakoski et al., 2015; Coumbe and Groarke, 2018). This imbalance may be a reflection of the decrease in catecholamines produced by high physiological stress, systemic inflammation, and reactive oxygen species (Hojman et al., 2018). Control of the patient’s autonomic imbalance could predict the risk of cardiotoxicity and cardiovascular disease in this population (Arab et al., 2016).

Previous exercise interventions have attempted to restore the autonomic balance of breast cancer patients (Lavín-Pérez A. M. et al., 2021). Although the interventions carried out are highly heterogenous, supervised exercise interventions involving strength training and cardiovascular exercise seem to have promising results in increasing overall HRV (the standard deviation of the interbeat interval of normal sinus beats, SDNN) and variables related to the parasympathetic branch (root mean square of successive differences between normal heartbeats, rMSSD), thus restoring the autonomic balance of breast cancer patients (Shin, Yang, and Kim, 2016; Dias Reis et al., 2017a; Mostarda et al., 2017; Toohey et al., 2020). However, there is a lack of continuous daily HRV assessment to monitor possible effects and alterations in this population.

Daily HRV assessment is widely used and particularly relevant to monitor the effects and control the exercise volume and intensity in the field of sport performance (Lundstrom, Foreman, and Biltz, 2022). However, there is a lack of studies focused on the usefulness of daily assessed HRV to monitor adaptations during a exercise program of cancer patients. Studies conducted on high-performance athletes and healthy individuals have demonstrated that HRV can provide objective measures of post-exercise recovery and indicate the presence of physiological stress or potential maladaptive states (Lundstrom, Foreman, and Biltz, 2022; Mosley and Laborde, 2022). Among others, HRV may provide information of exercise recovery and tolerance showing decreases of RMSSD and high-frequency (HF) measures in healthy individuals 24 h after a high-intensity session, while endurance training may have different effects (Mosley and Laborde, 2022). Moreover, HRV measures can provide critical information in breast cancer survivors including out-of-range values or large changes that may be related to the presence of certain symptoms such cancer-related fatigue (Crosswell et al., 2014; Shih et al., 2021), the accentuation of joint pain caused by treatments (Laroche et al., 2017; Forte et al., 2022), incubation or recuperation from common diseases (Khosravi et al., 2019; Frasch and Martin, 2022), or even psychological stress arising from personal or medical issues (Kim et al., 2018).

Based on the existing literature, there is a need of studies aimed to demonstrate the effectiveness of physical exercise in enhancing autonomic balance, as well as to examine the progression of daily HRV during the survivorship phase in women who participate in physical exercise interventions. Through daily analysis by employing useful measure tools such as photoplethysmography (Plews et al., 2017), potential imbalances and changes over time may be explored to obtain information about acute recovery from exercise and its evolution with training along time. Therefore, the goal of this study was to analyze the effects of a 16-wk exercise intervention on the autonomic modulation of breast cancer survivors. Moreover, the study also aimed to examine the evolution of daily measured HRV and the interaction with exercise sessions in this population.

## 2 Materials and methods

### 2.1 Participants, recruitment, and ethical procedures

Participants were randomly assigned to two groups, the exercise group (EG) and the control group (CG). The randomization and blinding allocation procedures were completed before informing the patients about the study. The participants were informed about the study details by the oncology team and the exercise professional, including the potential benefits and risks associated with exercise. A total of 34 patients accepted to participate and met the inclusion criteria at the Marqués de Valdecilla University Hospital in Santander (Cantabria).

Moreover, prior to conducting the study, the sample size was calculated utilizing G*Power software (3.1.9.5) (Kang, 2021). Due to there were no previous research assessing daily HRV in cancer patients, sample size calculation was conducted based on data from the study performed by Dias Reis et al. (2017b). Thus, taking as input information the effect size reported in that previous study, an alpha error probability of 0.05, and a power (1-beta error probability) of 0.85, a total sample size of 26 (13 patients in each of the groups) were needed.

**FIGURE 1:**
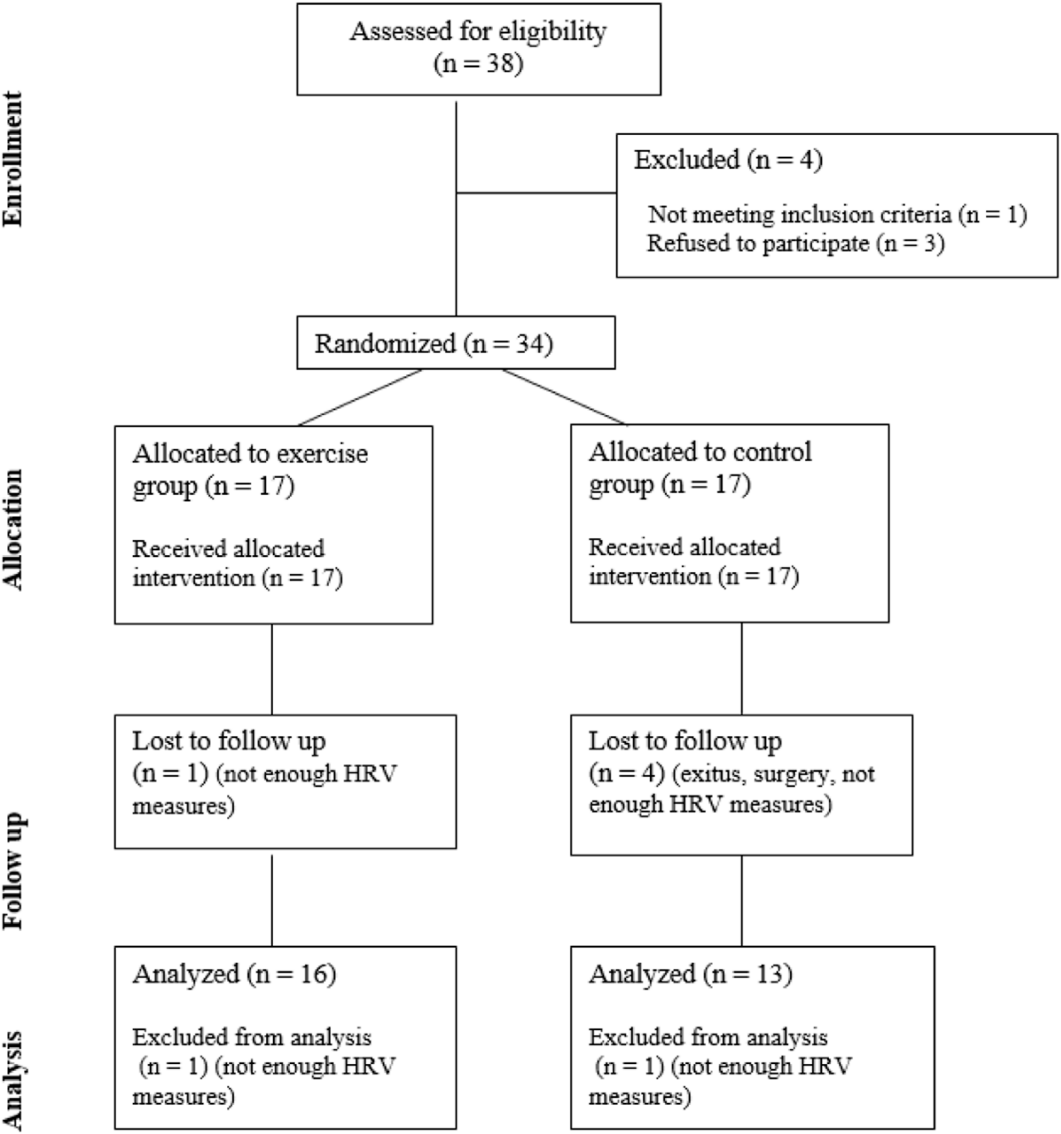
Flow diagram of the participants’ recruitment and enrollment process following the Consolidated Standards of Reporting Trials guidelines.

The following inclusion criteria were applied: (a) had been treated with radiotherapy and chemotherapy for breast cancer between 1 and 5 months before the study, (b) were between 18 and 65 years old, (c) and had not suffered from any other previous cancer. The oncology team set these criteria to ensure that patients were similar in terms of hospitalization and to enable the timely recovery of patients after they finished their in-hospital treatments. Additionally, those who did not accept to participate or were diagnosed with cardiac abnormalities diagnosed by echocardiogram at baseline were excluded. Written informed consent was obtained from the patients. During the investigation, those who developed cancer again, died (exitus), had a surgery, or did not measure at least 4 times per week to obtain sufficient data for analysis were excluded (refer to Figure 1 for further details).

The study design strictly followed the Helsinki Declaration of 2014 (Association General Assembly of the World Medical, 2014). The research adhered to ethical standards, which were approved by Rey Juan Carlos University (approval number: 1901202103121), and the Valdecilla Health Research Institute (IDIVAL) at the University Hospital of Marqués de Valdecilla (register identification number: 2021.214).

### 2.2 Study design: exercise group and control group

**FIGURE 2:**
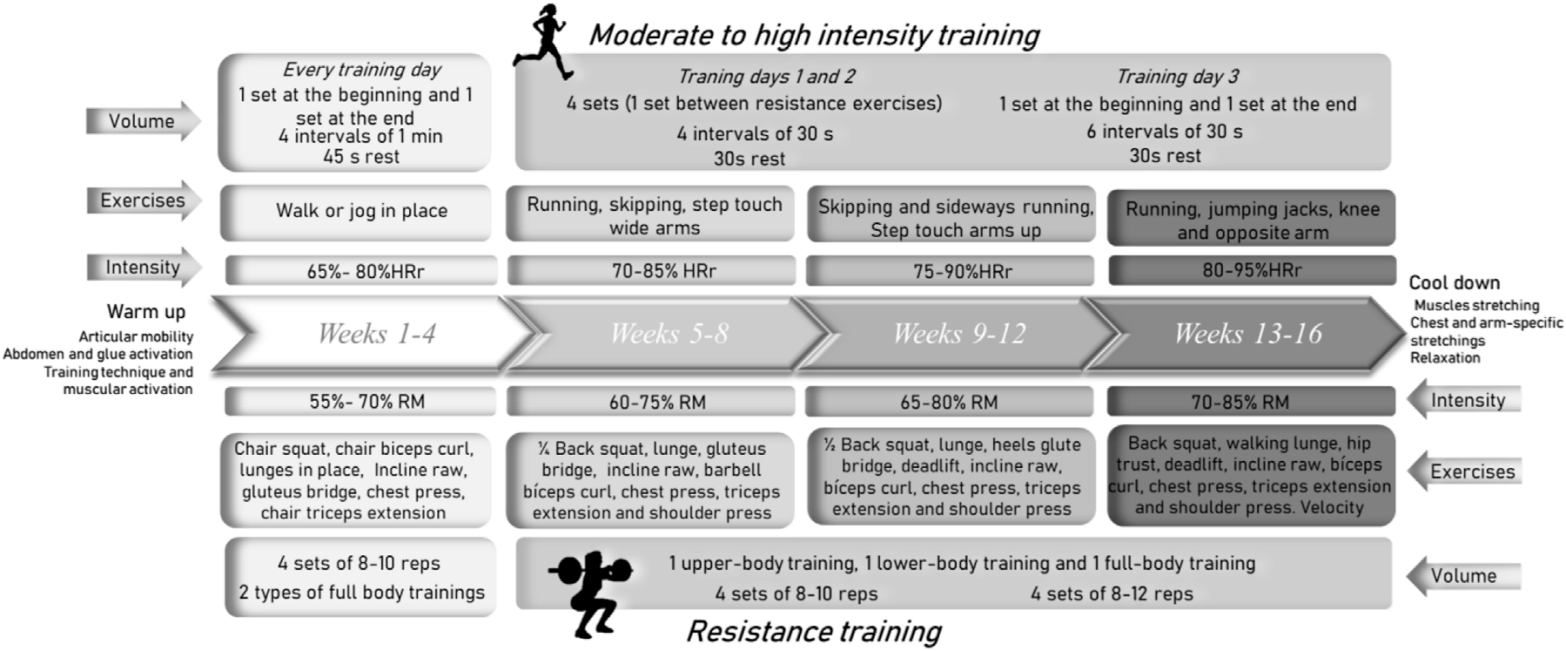
Exercise training program: volume, exercises and intensity characteristics.

Exercise group participants underwent to a home-based exercise program where all sessions were supervised through an online meeting with participants. The program included resistance and moderate-to-high intensity training and consisted of three sessions per week, including both strength and cardiovascular exercises in each session. All the details about the training program concerning set, repetitions, exercises, rests and intensity can be found in Figure 2. During the first 4 weeks of the program, participants underwent the mesocycle aimed to familiarize them with the training and make neuromuscular adaptations. In the following weeks, from week 5 to week 16, two microcycles were developed increasing the technical complexity of the exercises. Regarding the intensity of the intervention workouts, it progressed by 5% every 4 weeks in both strength or cardiovascular component exercises (Kirkham et al., 2018). The sessions were conducted online with direct supervision, and controlling the heart rate in real time, from a fitness trainer specialized in physical exercise for cancer patients.

The initial intensity of each participant in strength exercises (back squat, deadlift, lunge, bench press, shoulder press, biceps curl) was calculated by an initial evaluation indirectly measuring the maximum weight each patient could lift in one repetition (1RM) using an encoder (Chronojump Boscosystem, Spain). The tests were conducted on a multipower machine with a counterweight mechanism to ensure rectilinear movement and eliminate the impact of the barbell weight. Participants performed three warm-up repetitions using the barbell and added incremental weight discs based on their perceived effort and the decline in execution speed. The patients’ perceived exertion was recorded using the modified Borg scale, and weights were increased when the value drops to 2/10. Both in the evaluation and during sessions, exercises were performed using a training bar and weight discs (a multipower to guide the movement at the initial evaluation and a 2 kg bar with different weight discs during the intervention).

For cardiovascular training prescription, each patient’s was evaluated with the Modified Bruce Test (Bruce, 1974) on a treadmill to asses cardiorespiratory capacity, maximum heart rate, and patients’ perceived exertion. The test was done with a Polar H10 chest strap to monitor heart rate while the speed and incline of the treadmill increase every 3 min until the patient decides to stop. Sessions’ target intensity was prescribed based on the maximum heart rate using Karvonen’s formula (HRreserve) (Karvonen, 1957).

The participants assigned to the control group participated in the pre- and post-intervention assessments and measured their HRV daily. They were instructed to maintain their regular activities of daily living without any restrictions on exercise.

### 2.3 Measurements and outcomes

#### 2.3.1 Clinical and socio-demographic information

In both groups, different information about the characteristics of the participants was collected. To obtain sociodemographic information, data of the age and ethnicity of the patients were collected. As for clinical information, relevant information about their cancer and the treatment administered was archived. Specifically, the type of breast cancer, affected breast(s), type of chemotherapy treatment received, months since completion of chemotherapy, duration of radiotherapy and treatment administered during the study were collected.

#### 2.3.2 Variables and measurement of heart rate variability

The data for HRV analysis were measured by each patient by employing the HRV4Training software. The daily assessment was performed with photoplethysmography every morning while lying down upon awakening. The application has been previously validated (Plews et al., 2017) and used in multiple research studies to analyze exercise recovery and physiological stress (Altini and Plews, 2021).

The procedure was carried out in the following steps: 1) the application was installed during initial in-person physical evaluations performed for load and intensity establishment to teach patients to use the application and check the compatibility of their devices ensuring quality measurements. 2) A visual explanatory manual was provided to each patient to ensure the correct use of the application. 3) Before the start of the investigation, a familiarization period was provided, during which the coach checked daily measurements to provide feedback or assistance as needed. 4) In the investigation period, upon walking up, in a supine position, subjects picked up their mobile phones and assumed a supine position for 5 min. During this time, the smartphone camera was placed with the flashlight on the left index finger to capture a signal for 1 min (Plews et al., 2017). Patients were mentioned to go to the toilet or get up if they needed to do so, but then to lie back down in bed and after 5 min of relaxation to take the measurements. 5) Once the measure was complete, if rMSSD data was considered inadequate due to user error (e.g., finger movement over the camera), subjects were notified and asked to perform a new recording until obtaining a correct measurement. 6) Finally, a response indicating the correct measurement was received and the subjects’ application stored information for automatic transmission to researchers.

The processing signal phases consisted of averaging the red, green, and blue channels across the entire frame before converting it to the HSV (Hue, Saturation, Value) color space (Altini and Oliver, 2018). Next, the signal intensity component was filtered using a Butterworth bandpass filter with a passband frequency between 0.1 and 10 Hz to remove noise and the direct current (DC) component of the signal while preserving the alternating current (AC) component. The signal was sampled between 30 and 180 Hz using cubic spline interpolation to increase the resolution of HRV feature calculation. A slope inversion algorithm was used for peak detection, and peak-to-peak intervals were corrected for artifacts by removing intervals that differed from the previous interval by more than 20% (Plews et al., 2017; Altini and Oliver, 2018).

From the daily data collected from patients during the 16-week intervention period, measurements were categorized according to the respective intervention groups. Specifically, the EG measurements were classified as follows: pre-training assessments, assessments conducted on the morning of the training day (Day 0), measurements taken 24 h post-exercise, measurements taken 48 h post-exercise, and measurements taken 72 h post-exercise. Meanwhile, for the CG, four non-weekend days were selected to establish reference measurements for each patient (designated as measurements 1, 2, 3, and 4), which could be compared to those taken by the experimental group.

Within the variables gathered through the utilization of the mobile application, two key metrics were extrapolated: rMSSD and SDNN. The rMSSD measure encompasses the variance in the temporal intervals between successive normal-to-normal heartbeats (Shaffer and Ginsberg, 2017). It serves as an indicator of the fluctuation in the functioning of the parasympathetic nervous system, which governs the body’s relaxation response (Shaffer and Ginsberg, 2017). The computation of this metric involves extracting the square root of the mean of the squared differences observed between consecutive normal-to-normal intervals. Conversely, both sympathetic nervous system and parasympathetic nervous system contribute to SDNN metrics encapsulates the overall variability in the temporal intervals between consecutive heartbeats, encompassing normal-to-normal sinus beats (Shaffer and Ginsberg, 2017). The calculation of the SDNN metric involves determining the standard deviation of all normal-to-normal intervals recorded over a specified period (Shaffer and Ginsberg, 2017).

To make the selected variables, rMSSD and SDNN, comparable between patients, the neperian logarithm was calculated to obtain lnrMSSD and lnSDNNN, as previous investigations have performed (Moya-Ramon et al., 2022). To calculate the evolution of the patients during the 16 weeks of intervention, the average of each variable was calculated for each measurement time (EG: Day 0, 24 h post-exercise, 48 h post-exercise and 72 h post-exercise; CG: measurement 1, 2, 3 and 4) for each month (month 1, month 2, month 3 and month 4). Subsequently, following the methodology performed by previous investigations (Iglesias-Soler et al., 2015; Flatt et al., 2019; Estévez-González et al., 2021), to analyze the change produced, the percentage change of each month with respect to month 1 was calculated for each measurement time.

That is, for example, to know the % change of lnSDNN in month 3 in the measurement at 48 h post-exercise, in the case of the EG, the mean of lnSDNN 48 h post-exercise of month 3 was calculated, the result was then subtracted from the mean lnSDNN 48 post-exercise, the lnSDNN 48 post-exercise month 1 and the result was divided by the mean lnSDNN 48 post-exercise month 1 and multiplied by 100 ([[lnSDNNN_48 h_M3-lnSDNN_48 h_M1]/lnSDNNN_48 h_M1]*100).

### 2.4 Statistical analysis and calculations

After completing the necessary calculations in Microsoft Excel, the resulting data were imported into IBM SPSS Statistics version 28 (Statistics and IBM, 2021) for analysis. To evaluate the potential interactions inter-groups, recovery times, and months, repeated-measures analysis of variance (ANOVA) with Bonferroni adjustments were employed for each variable. In the statistical analysis, statistical significance as having an alpha level of 0.05 was established. Furthermore, partial eta squared was utilized (ηp^2^) to determine the fraction of the total variance of the response variable explained by a specific predictor in the model. To interpret the effect size the following classification system was followed: small (0.01), medium (0.06), and large (0.14) (Cohen, 2013).

## 3 Results

### 3.1 Participants’ demographic and clinical characteristics

A total of 29 participants (EG: 16 and CG: 13) completed the study. Their mean age was 50 (SD = 7.24) ranging from 38 to 63 years. Within the breast cancer types included in the study, most of the patients had luminal cancers (24.14% with phenotype A and 55.17% with phenotype B) and 20.69% were diagnosed as triple negative. All participants were treated with adjuvant (65.51%) or neoadjuvant (34.48%) chemotherapy and radiotherapy. Detailed information for each group can be found in Table 1.

**TABLE 1:** Clinical and demographic information for each intervention group.

	Exercise Group	Control Group	*p*-value (*t*-test)
Sample size	16	13	
Ethnicity (%)			
Caucasian	100	84.61	
Hispanic	0	5.18	
Mean age (SD)	50.63 (8.16)	49.59 (6.1)	0.383
Mean heart rate in rest (SD)	68.94 (4.34)	69.73 (3.78)	0.741
Mean systolic blood pressure (SD)	117.81 (17.34)	120.21 (15.85)	0.606
Mean diastolic blood pressure (SD)	75.19 (11.34)	76.82 (12.42)	0.762
Breast cancer type (%):			
Luminal A phenotype	25.00	23.08	0.107
Luminal B phenotype	56.25	53.85	0.678
Triple Negative	18.75	23.08	0.297
Chemotherapy treatment timing (%):			
Neoadjuvant	31.25	38.46	0.482
Adjuvant	68.75	61.54	0.482
Treatments during the intervention (%):			
Tamoxifen/Zoladex/Exemestane	50.00	46.15	0.296
Letrozol	31.25	30.77	0.325
None	18.75	23.08	0.452
Mean of weeks since the end of radiotherapy (SD)	12.5 (5.5)	11.23 (5.2)	0.517

SD, standard deviation.

### 3.2 Inter-group differences in heart rate variability

**FIGURE 3:**
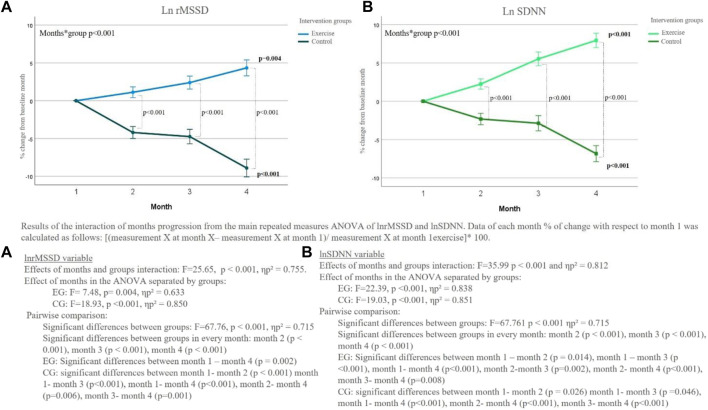
% of change of LnrMSSD and lnSDNN % from month 1 of exercise and control groups during the intervention (including the average of each recovery time).

As shown in Figure 3, during the intervention the groups became increasingly differentiated along months. In this regard, significant differences were obtained in the interaction between months and groups in both lnrMSSD (F value = 25.65, *p* < 0.001 and ηp^2^ = 0.755), and lnSDNN (F value = 35.99, *p* < 0.001 and ηp^2^ = 0.812). Therefore, the exercise group significantly increased their HRV variables compared to the control group across the 4 months. The pairwise analyses showed that changes for the exercise group were significantly different compared to the control group, in each of the months. The exercise group showed an increase in HRV variables as a result of the exercise, while the control group demonstrated a reduction.

### 3.3 Main heart rate variability changes in each group

The exercise group showed significant improvement month-to-month in both the lnrMSSD variable (*F* = 7.48, *p* = 0.004, ηp^2^ = 0.633) and lnSDNN (F = 22.39, *p* < 0.001, ηp^2^ = 0.838), with the variables progressively increasing as months passed (Figure 3). Pairwise analysis showed significant differences in lnrMSSD between month 1 and month 4 (*p* < 0.001) and between month 2 and month 4 (*p* = 0.006). For lnSDNN, all pairwise comparisons between months were significant (*p* ≤ 0.05).

On the other hand, the control group showed a gradual decrease in lnrMSSD and lnSDNN values (Figure 3). The effect of months was statistically significant for lnrMSSD (*F* = 18.93, *p* < 0.001, ηp^2^ = 0.850), with differences between all months except month 2 and month 3. Similar effects were observed in lnSDNN for the months (*F* = 19.03, *p* < 0.001, ηp^2^ = 0.851), with statistically significant differences between month 1 and month 2 (*p* = 0.029), month 1 and month 4 (*p* < 0.001), month 2 and month 4 (*p* = 0.015), and between month 3 and month 4 (*p* = 0.004).

### 3.4 Exercise group heart rate variability at each recovery time

**FIGURE 4:**
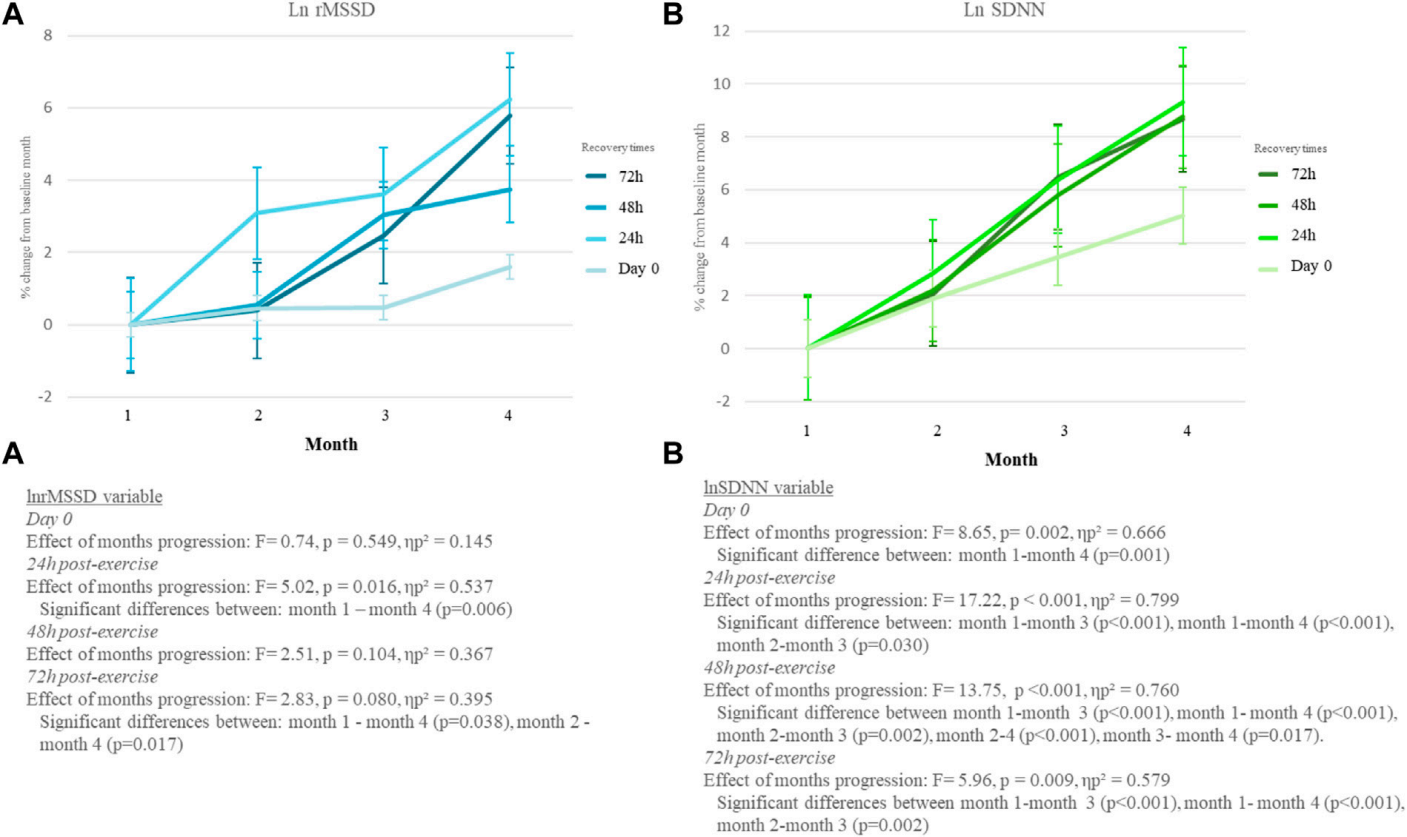
% of change of LnrMSSD from month 1 of exercise and control groups during the intervention in each recovery time.

Figure 4 shows how patients were able to adapt to exercise over the course of the months. Accordingly, each recovery time (day 0, 24 h after exercise, 48 h after exercise, and 72 h after exercise) for the exercise group was showed.

The initial measurement was taken by patients before they trained and was named in this article as “Day 0”. The progress of months showed a significant effect on lnSDNN_Day0_ (*F* = 8.65, *p* = 0.002, ηp^2^ = 0.666) but not in lnrMSSD_Day0_ variable (*F* = 0.74, *p* = 0.549, ηp^2^ = 0.145) (presented in Figure 4). Moreover, in the pairwise comparison lnrMSSD_Day0_ did not achieve significant differences among months (*p* > 0.05) while lnrMSSD_Day0_ did between month 1 and month 4 (*p* = 0.001).

Furthermore, exercise group data 24 h post-exercise shows the measurements that the exercise group patients took the morning after training (24 h post-exercise). In this case, the course of months had a significant interaction in the lnrMSSD_24h_ (*F* = 5.02, *p* = 0.016, ηp^2^ = 0.537) and lnSDNN_24h_ (*F* = 17.22, *p* < 0.001, ηp^2^ = 0.799) variables. In addition, differences were obtained only between month 1 and month 4 in the lnrMSDD_24h_ variable (*p* = 0.006). Whereas for the lnSDNN_24h_ variable, there were significant differences between month 1 and month 3 (*p* < 0.001), month 1 and month 4 (*p* < 0.001), month 2 and month 3 (*p* = 0.030).

The results taken in the morning 48 h after exercise sessions achieved a significant effect of the interaction of the months in the lnSDNN_48h_ variable (*F* = 13.75, *p* < 0.001, ηp^2^ = 0.760) but not in the lnrMSSD_48h_ variable (*F* = 2.51, *p* = 0.104, ηp^2^ = 0.367). Specifically, the pairwise comparison showed, only the lnSDNN_48h_ variable a significant effect of the months (*F* = 13.75, *p* < 0.001, ηp^2^ = 0.760) with significant differences between months 1 and 3 (*p* < 0.001), 1 and 4 (*p* < 0.001), 2 and 3 (*p* = 0.002), 2 and 4 (*p* < 0.001), and 3 and 4 (*p* = 0.017). In lnrMSSD_48h_ not significant differences between moths were reached (*p* > 0.05).

Finally, also Figure 4 shows the measurements results taken from exercise group patients in the morning 72 h after training sessions. In this sense, results showed a significant interaction between months both variables (lnrMSSD_72h_: F = 2.83, *p* = 0.080, ηp^2^ = 0.395; lnSDNN_72h_: F = 5.96, *p* = 0.009, ηp^2^ = 0.579). Moreover, significant differences between months were observed between months 1 and 4 (*p* = 0.038) and 2 and 4 (*p* = 0.017) in the lnrMSSD_72h_ variable, and between months 1 and 3 (*p* < 0.001), 1 and 4 (*p* < 0.001), 2 and 3 (*p* = 0.002) in the lnSDNN7_2h_.

## 4 Discussion

The main aim of the study was to analyze the differences between exercise and control groups in the autonomous nervous system activity, through two selected HRV indexes (lnrMSSD and lnSDNN) during a 16-wk exercise intervention, examining both inter-group and intra-group differences over time, as well as the adaptation of the recovery response to training load after of every exercise session. In this regard, the results obtained showed a significant inter-group and between months interaction along time achieving a significant enhancement in the exercise group compared to the control group in both lnrMSSD and lnSNDD. Furthermore, significant within-group differences between the months in each recovery time for lnSDNN were observed while in the lnrMSSD variable. These results suggest that exercise may be an effective tool in improving parasympathetic activity and balance in sympathetic-parasympathetic activity. Findings from the current study are in line with previous pre-post investigations based in aerobic and resistance training (Shin, Yang, and Kim, 2016; Mostarda et al., 2017; Lee et al., 2019; Toohey et al., 2020). Despite the potential for exercise to induce stress upon the body (Hanns et al., 2019), gradual and progressive implementation may serve to counteract such effects by reducing the physiological stress resulting from treatments. Physical exercise may play a crucial role in promoting the activation of the parasympathetic nervous system, through the possible increase in availability of nitric oxide and suppression of angiotensin II (Kruk, Kotarska, and Aboul-Enein, 2020). This may then lead to the activation of mitochondria, the NADPH system, neutrophils, phagocytes and catecholamines, regulating the concentration of reactive nitrogen and oxygen species (Hojman et al., 2018).

Due to the increased access and use of technology by the majority of people, home-based exercise programs have emerged as a strategy to reduce costs and reach a larger population when resources allocation is a limiting problem. The current study shows that benefits can be achieved through a clinical intervention carried out in non-clinical environment in a completely safe and supervised manner, conducted through online meetings with patients and with the initial recommendation of the oncologist (Hardcastle and Cohen, 2018). In contrast with other oncological home-based interventions were participants make the recommended exercise on their own or this periodic supervision strategies (Hanson et al., 2023; Pelosi et al., 2023), the current exercise program was completely supervised in real time. For this purpose, both the participants and the trainer had their phone or computer cameras switched on and participants’ heart rate was monitored and controlled in real time by the trainer. In this regard, the supervision of all sessions could be a key factor to optimize the benefits (Newton et al., 2018), based on the results of a previous meta-analysis comparing the effects of supervised and unsupervised home-based exercise interventions in quality of life and functional capacity (Kraemer et al., 2022).

Regarding home-based interventions, it is mandatory to move beyond generalized exercise recommendations or low-intensity exercises that are basic and unsupervised (Adams et al., 2018; Lopez et al., 2018; Newton et al., 2018). Although it is widely-known that almost any kind of physical activity is better than physical inactivity, the type of exercise and the characteristics in terms of intensity, volume, rests, etc. must be carefully designed to maximize the benefits and induce catecholamine secretion and binding to receptors, contributing to a positive effect on cellular immune responses in cancer cells, inflammation, angiogenesis, tissue invasion, and epithelial to mesenchymal transformation (Dethlefsen et al., 2017; Kruk, Kotarska, and Aboul-Enein, 2020). A higher level of activity in the vagal nerve may have an inhibitory effect on inflammation in the tumor microenvironment and disrupt the mutual influence between tumor cells and the surrounding inflammatory microenvironment (Zhou et al., 2016). Therefore, it is suggested that regular moderate to high-intensity training may be necessary to observe significant changes in variables such as lnrMSSD and lnSDNN, in accordance with numerous studies (Kruk, Kotarska, and Aboul-Enein, 2020) and the present study’s results.

One of the primary findings of this study pertains to the behavior of the lnrMSSD and lnSNDD variables in the control group over the investigation months. Notably, a clear reduction in these variables was observed, with medium to large effect sizes. The decrease in the lnSNDD variable, in particular, may indicate a high imbalance between the sympathetic and parasympathetic branches indicating worse health and physiological resilience, which are negatively correlated with cardiac risk (Shaffer and Ginsberg, 2017). The decline in lnrMSSD, or rMSSD, is related to a reduction in parasympathetic activity and has been investigated as a survival prognostic variable (Kloter et al., 2018). These variables can serve as an indicator of autonomic dysfunction which, could be caused by treatments cardiotoxicity (Coumbe and Groarke, 2018). However, these measurements performed in the present article do not include other calculations that could add very relevant information about the degree of modulation due to sympathovagal influence. This is the case of the Autonomic Nervous System Index (ANSI) (Sala et al., 2017), which could provide information about the autonomic performance of the patients, as other research studies have done in breast cancer patients (Lucini et al., 2019; Lucini et al., 2020). To do this, the inclusion of variables and measurements that the present research could not obtain with the measurement protocol carried out would be necessary. Thus, future research could delve into the methodology and obtain more robust results that can support the findings of this study.

Moreover, the results found are consistent with previous research where breast cancer patients present significantly lower values of rMSSD, HF (high frequency), and SDNN 1 year after treatment (Caro-Moran et al., 2016). Therefore, the role of the parasympathetic nervous system and its evolution is crucial in monitoring the health of patients, as its measuring variables, such as HF, are directly correlated with survival (Giese-Davis et al., 2015). Likely, this mechanism occurs progressively, and thus, patients may continue to suffer the consequences of prior treatments and physical inactivity. Additionally, it is important to note that many of the patients were receiving hormonal aromatase inhibitors that can cause fatigue and joint pain, both of which can further exacerbate their deconditioning (Crosswell et al., 2014; Laroche et al., 2017).

Beyond the difference in autonomic control between the group of patients who exercised and those who did not, the present study was focused on the progression in each recovery time after the training sessions. In this regard, the results showed that lnrMSSD and lnSDNN are sensitive to exercise training from the first month and may be used as physiological data to assess how each patient endures and adapts to the suggested exercise intensity. Therefore, unlike previous pre-post studies carried out (Shin, Yang, and Kim, 2016; Dias Reis et al., 2017a; Mostarda et al., 2017), the present study analyze and report a differentiating quality component, providing data on the progressive HRV changes that patients experience. This information allows the researcher or trainer to monitor and control the physiological adaptations caused by exercise (and other sources) before and after each session. Therefore, HRV may be considered as a physiological variable that may early reflect biological or psychological health or wellbeing and may help to tailor the exercise sessions regarding volume, intensity, or loads.

Although the current study provides solid findings about the beneficial effect of exercise in modulating the autonomic system and the useful of monitor daily HRV some limitations of the research must be noted. First, the sample size is not large, and various participants declined to take part, which may limit the generalization results. Moreover, during the intervention, some participants in the control group were lost and the information of some patients could not be used because they did not take enough measurements. Furthermore, the patients included in the study had suffered from different types of breast cancer, which makes the sample somewhat heterogeneous, especially when considering the treatments that some of them continued to take during the intervention. Future studies could replicate the study with larger sample size to confirm the results when monitoring daily autonomic response. Moreover, it would be interesting to explore the possibility of prescribing exercise intensity regarding patients daily parasympathetic measures in breast cancer survivors.

## 5 Conclusion

Physical exercise may serve as an effective tool in the prevention of the reduction of lnrMSSD and lnSDNN in breast cancer patients. In the best of our knowledge, the current study is the first one aimed to evaluate the day-to-day variations of HRV to every session of a 16wk exercise intervention in breast cancer patients. Results revealed that while participants who performed a training protocol improved their parasympathetic control, patients from the control group experienced a significant reduction that may indicate a high imbalance between the sympathetic and parasympathetic branches indicating worse health and higher cardiac risk. Moreover, the present investigation shows the relevance of lnrMSSD and lnSDNN to monitor the adaptations or inadaptations of patients to exercise as well as to register and control the progress in the recovery adaptations.

## Data Availability

The raw data supporting the conclusion of this article will be made available by the authors, without undue reservation.
